# Managing Anemia: Point of Convergence for Heart Failure and Chronic Kidney Disease?

**DOI:** 10.3390/life13061311

**Published:** 2023-06-01

**Authors:** Oana Nicoleta Buliga-Finis, Anca Ouatu, Daniela Maria Tanase, Evelina Maria Gosav, Petronela Nicoleta Seritean Isac, Patricia Richter, Ciprian Rezus

**Affiliations:** 1 Department of Internal Medicine, “Grigore T. Popa” University of Medicine and Pharmacy, 700115 Iasi, Romania; oana_finish@yahoo.com (O.N.B.-F.); dr.evelinagosav@gmail.com (E.M.G.); isac_petronela@yahoo.com (P.N.S.I.); ciprian.rezus@umfiasi.ro (C.R.); 2 Internal Medicine Clinic, “Sf. Spiridon” County Clinical Emergency Hospital, 700111 Iasi, Romania; 3 Department of Rheumatology and Physiotherapy, “Grigore T. Popa” University of Medicine and Pharmacy, 700115 Iasi, Romania; patriciarichter12@yahoo.com; 4 Rheumatology Clinic, Clinical Rehabilitation Hospital, 700661 Iasi, Romania

**Keywords:** cardiorenal syndrome, anemia, HIF-PH inhibitors, erythropoiesis-stimulating agents, iron therapy, hepcidin antagonists

## Abstract

The pathologic triangle formed by chronic heart failure (HF), chronic kidney disease (CKD), and anemia carries high morbidity and mortality rates and decreases quality of life. Anemia represents a common condition in patients with advanced HF and CKD, with a total prevalence in cardiorenal syndrome (CRS) ranging from 5% to 55%. Searching for a pragmatic approach for these patients with guided and disease-specific recommendations beyond just targeted hemoglobin therapeutic behavior represents the core of research for ongoing clinical trials. It is well known that the prevalence of anemia increases with the advancement of CKD and HF. The physiopathological mechanisms of anemia, such as the reduction of endogenous erythropoietin and the decrease in oxygen transport, are leading to tissue hypoxia, peripheral vasodilation, stimulating neurohormonal activity, and maintenance of the progressive renal and cardiac dysfunction. Given the challenges with the treatment options for patients with cardiorenal anemia syndrome (CRSA), new therapeutic agents such as hypoxia-inducible factor–prolyl hydroxylase domain inhibitors (HIF-PH) or hepcidin antagonists are emerging in the light of recent research. This review summarizes the potential therapeutic tools for anemia therapy in the cardiorenal population.

## 1. Introduction

Cardiorenal syndrome (CRS), first described in 1836 by Robert Bright, includes hemodynamic interactions of the heart and kidney in which acute or chronic dysfunction in one organ may induce acute or chronic dysfunction in the other organ [1]. 

Based on the primary organ dysfunction and type (acute or chronic), there are five cardiorenal syndromes: type 1 (acute cardiorenal syndrome), type 2 (chronic CRS), type 3 (acute renocardiac syndrome), type 4 (chronic renocardiac syndrome), and type 5 (secondary CRS) [2].

The heart and kidney interaction involves complex hemodynamic, biochemical, and hormonal pathways: renin-angiotensin-aldosterone system (RAAS) overactivation, sympathetic nervous system (SNS) dysfunction, hemodynamic alteration, oxidative stress, fibrosis, chronic inflammation, mitochondrial dysfunction, and anemia [3]. The triad of anemia, HF, and CKD, or “cardiorenal anemia syndrome” (CRAS), has complex and numerous pathophysiological mechanisms, and the lack of specific recommendations for the management of cardiorenal patients with anemia has been the leading motor for recent studies [4].

Anemia in cardiorenal syndrome is multifactorial, and various mechanisms are implicated: the use of renin–angiotensin–aldosterone system inhibitors that leads to decreased erythropoietin synthesis; low cardiac output that is responsible for bone marrow hypoperfusion; the inflammatory status that causes erythropoietin resistance; iron deficiency, hemodilution, and gastrointestinal blood losses [5].

The definition of anemia according to the World Health Organization is a concentration of hemoglobin <13.0 g/dL in men and <12.0 g/dL in women [6].

Iron deficiency is defined by current clinical guidelines as a serum ferritin concentration <100 ng/mL or a serum ferritin <300 ng/mL with transferrin saturation <20% [7]. For patients with anemia without iron therapy or for patients who need erythropoiesis-stimulating agents, a transferrin saturation ≤30% and serum ferritin ≤500 ng/mL are indicated by KDIGO guidelines as setting points for iron supplementation [8].

In this review, we aim to discuss the main physiopathological aspects of anemia in chronic kidney disease and heart failure. Considering that the treatment of anemia in the cardiorenal population is difficult and complex and needs individualized approaches, we have highlighted the main therapeutic options supported by recent studies. Hypoxia-inducible factor–prolyl hydroxylase domain inhibitors (HIF-PH), as new therapeutic agents, are promising tools for anemia management in patients with both CKD and HF. We emphasized details about the mechanism of action and pharmacological properties of this therapeutic class, as well as the existence of new possible therapies.

## 2. Epidemiology

Anemia of chronic diseases represents a common condition in patients with advanced heart failure and chronic kidney disease [9]. The prevalence of anemia rose from 9% to 79% in patients with HF according to the New York Heart Association (NYHA) functional class classification and from 21% to 70% in patients with CKD. Comparing newly diagnosed HF with congestive HF, the incidence of anemia was 17% to 56% in the second group of patients [10]. Furthermore, being a common complication in CKD, anemia is more severe as the kidney function declines, with 8.4% prevalence at stage 1 and 53.4% prevalence at stage 5. 

The prevalence of newly diagnosed anemia in the observational study NADIR-3 for patients with CKD stage 3 without anemia ranged from 11% in the first year to 26% in the third year of observation. Anemia in these patients was associated with an increase in mortality (10.3 vs. 6.6%), higher rates of hospitalizations (31.4 vs. 16.1%), cardiovascular events (16.4 vs. 7.2%), and progression of CKD to stages 4–5 [11].

For non-dialysis patients, anemia (defined as a hemoglobin level below 13 g/dL in men and below 12 g/dL in women) has a prevalence between 30 and 40% [12].

Several studies suggest that anemia and kidney dysfunction have an important impact on HF patients. For example, data from a population of more than one million patients show a mortality risk for two years that increased from 27% in patients with HF to 35% in those with HF and anemia, 38% in those with both chronic kidney disease and heart failure, and 46% in the cardiorenal anemia syndrome [5]. 

## 3. Physiopathology of Anemia in Cardiorenal Syndrome

The reduction of endogenous erythropoietin, impaired iron absorption, increased hepcidin levels, increased levels of uremic toxins that cause a reduced bone marrow response to EPO, systemic inflammation conditions, and vitamin deficiencies are some of the underlying mechanisms of anemia in CKD [13]. 

For CKD patients, hepcidin measurement can be used in the anemia diagnosis for both non-dialysis and dialysis patients. While low hepcidin and anemia indicate iron deficiency, anemia with high hepcidin levels reflects one of the following situations: hepcidin expression could not be inhibited due to inflammation or the presence of sufficient iron stores. Therefore, hepcidin is an important tool for the management of anemia in CKD patients. While in the first situation iron supplementation can be used, in the other two situations, ESA therapy (with or without additional iron supplementation) and anti-hepcidin agents are indicated [14].

The development of anemia in patients with decreased renal function is thought to be related to other comorbidities such as diabetes, where the EPO deficiency is a consequence of interstitial fibrosis, diabetic nephropathy, or glycation of the EPO receptor due to hyperglycemic status [15]. 

In HF, factors such as increased levels of interleukin-6 and tumor necrosis factor-α that act by decreasing EPO production, decreasing the release of iron from the reticuloendothelial system, and decreasing iron absorption from the gut, along with the effects of ACE inhibitors on the hematopoietic system, lead to anemia [16]. The use of ACE inhibitors decreases hematocrit levels, which lowers the circulating angiotensin II, further inhibiting the growth of erythroid progenitors and N-acetyl-seryl-aspartyl-proline catabolism, causing anemia in the first 3 months of therapy. Studies suggest that the effect is reversible after 3–4 months of interrupted administration.

**Figure 1:**
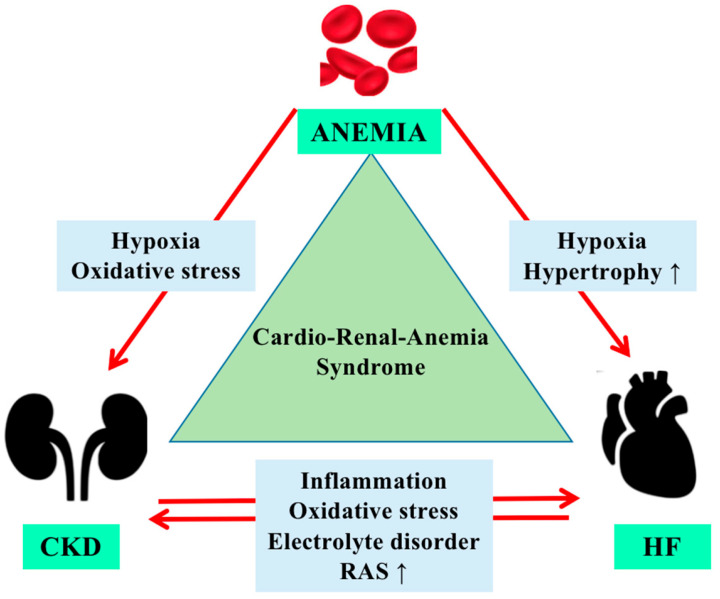
Cardiorenal anemia syndrome (CRSA) along with the main physiopathological mechanisms. CKD: chronic kidney disease; HF: heart failure; RAS: renin-angiotensin system. ↑ = increase.

Among the mechanisms that can produce anemia in HF are cited: nutritional deficiency, particularly iron deficiency; the reduced capacity of erythropoiesis secondary to the medular hypoperfusion; hemodilution; uremic gastritis; non-steroidal anti-inflammatory drug administration; anticoagulation and antiplatelet therapy, which can predispose to hemorrhagic accidents. Renal failure, in which EPO production is reduced, and the presence of proteinuria with possible loss of transferrin and EPO represent other causes of anemia (Figure 1) [17]. 

Hypoxia, as a secondary effect of anemia, increases inflammation and oxidative stress in CKD patients. Further, the reduced renal perfusion activates the renin-angiotensin system, changes that reduce cardiac output and contribute to the process of left ventricular hypertrophy. As in a vicious circle, these changes decrease organ perfusion (including the kidney), making cardiorenal-anemia syndrome a serious and complex disease [18].

The two cytokines (tumor necrosis Factor alpha (TNF α) and interleukin-6 (IL-6)) that are elevated in both CKD and HF due to the inflammatory status can determine several hematological disorders: reduced production of erythropoietin (EPO) in the kidneys, a reduced response of the bone marrow to EPO, decreased iron absorption from the gut, and a hepcidin-induced sequestration of iron in the macrophages. Hepcidin, which is released by IL-6 from the liver, inhibits ferroportin, a key protein that controls the release of iron from hepatocytes, the gastrointestinal tract, and macrophages into the circulation. This leads to a low serum iron level with adequate total iron stores but a diminished delivery of iron to the bone marrow. Because hepcidin has a renal metabolism, patients with CKD and those with HF in whom the mean creatinine clearance is <60 mL/min/1.73 m^2^ have increased levels that could explain the iron deficiency [19].

As chronic kidney disease progresses, hepcidin levels increase and, on the contrary, decline as heart failure advances, being associated with poor outcomes in HF. Chronic hypoxia secondary to lung congestion, which grows hypoxia-inducible factors expression, could represent a key explanation of low hepcidin levels in cardiac failure evolution [20]. 

Erythroferrone (ERFE) was recently discovered as a suppressor of hepcidin, as suggested in [21]. ERFE is secreted by erythroblasts in the marrow but also in extramedullary sites, contributing to iron delivery during stress erythropoiesis [22]. EPO deficiency and resistance represent two common characteristics of anemia in both CKD and HF. Serum EPO levels are higher in CKD and HF patients but disproportionately low for the degree of anemia [23]. 

Hypoxia-inducible factor (HIF) is a heterodimer with two subunits: HIF-α and HIF-β, their main roles being to reduce oxygen consumption and promote oxygen delivery [24,25]. Among the three isoforms of the α subunit, HIF-2α represents a key element in the hypoxic response, being involved in upregulating EPO gene expression in hypoxia [26]. 

Hypoxia-inducible factor 1 (HIF1) activates numerous genes, 4 of which are important for the effect of EPO: the nitric oxide synthase gene, which controls arterial blood pressure in the presence of the EPO pressure-effect; the transferrin gene, implicated in the transport of iron to erythroid cells; the tyrosine hydroxylase gene, and the vascular endothelial growth factor (VEGF) gene [27]. In anemic or hypoxic conditions, HIF1 activates the expression of the EPO gene [28]. Both HIF-1α and HIF-2α are implicated in atherosclerotic and hypertensive vascular disorders, contributing to the evolution and progression of CKD and HF. HIF-1α is responsible for promoting inflammation and hypertrophy in cardiomyocytes [29]. 

There is no information on how HIF is activated in HF, and there is also no data to support its quantification. Patients with heart failure may experience brief episodes of hypoxia, which may not sufficiently stimulate the production of EPO, leading to anemia [30].

HIF-1α is sensitive to environmental oxygen and is expressed in various cells, while HIF-2α is expressed selectively in endothelial cells in the heart, specialized peritubular interstitial cells in the kidney, alveolar epithelial cells in the lung, and hepatic parenchymal cells. Hypoxia upregulates the activities of HIF-1α and HIF-2α and inhibits the family of 3 prolyl hydroxylases (PHD1, PHD2, and PHD3) whose role is to degrade these two factors [29].

**Figure 2:**
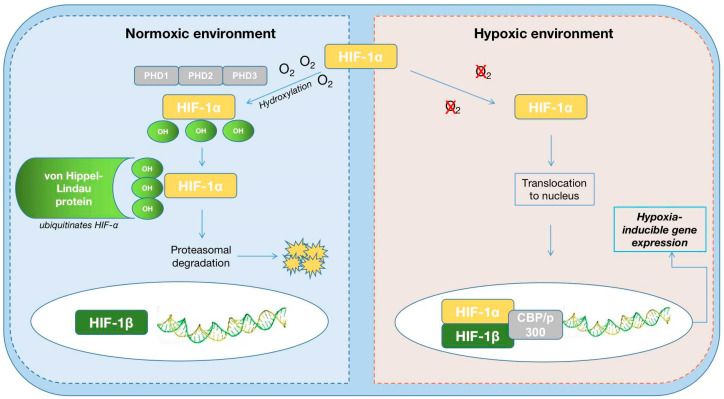
Hypoxia-inducible factor under normoxic and hypoxic conditions; HIF: hypoxia-inducible factor; O_2_: oxygen; OH: hydroxyl; PHD: prolyl hydroxylase domain protein; CBP: CREB-binding protein.

Hypoxia-inducible factor 1 is activated by hypoxia after phosphorylation at oxygen concentrations of approximately 4–5%, transactivating numerous genes that contribute to the final effect of EPO: the vascular endothelial growth factor (VEGF) gene, the tyrosine hydroxylase gene, the transferrin gene, and the nitric oxide synthase gene [31]. After the hydroxylation of HIF1α, the E3 ubiquitin ligase von Hippel Lindau (pVHL) promotes proteasomal degradation by binding to HIF1α (Figure 2). 

In hypoxic conditions, prolyl hydroxylase domain (PHD) enzyme action is prevented, and HIF1α is translocated to the nucleus, representing a possible target for hypoxia-inducible factor–prolyl hydroxylase inhibitors (HIF-PHIs) [32,33].

While HIF-1α stimulates the transcription of proteins that reduce oxygen consumption and boost angiogenesis, HIF-2α is the main inducer of erythropoietin synthesis.

Chronic kidney disease is characterized by upregulation of HIF-1α, but down-regulation of HIF-2α. HIF-2α deficiency likely explains why chronic kidney disease is accompanied by anemia and blunted production of erythropoietin, as well as why impaired erythropoietin synthesis is accompanied by increased inflammatory and angiogenic markers.

In cardiomyocytes, HIF-2α activation has protective effects. When HIF-2α expression is reduced, as in chronic cardiac disorder, the heart’s inflammasomes become activated [29]. 

By blocking PHDs, HIF prolyl hydroxylase inhibitors (HIF-PHIs) stabilize HIFs, enabling HIF-2α to act on EPO-producing cells in the kidney and liver to encourage endogenous EPO generation and subsequent hematopoiesis [34].

As a result, it is widely acknowledged that hypoxia plays a significant role in the development of CKD-HF, and that addressing hypoxia may represent a promising treatment approach for the cardiorenal anemia syndrome [35].

## 4. Managing Anemia in Cardiorenal Patients

The management of anemia in cardiorenal syndrome requires a complex approach that takes into account the following aspects: the classification of kidney and heart disease, the iron status, Hb levels, left ventricular ejection fraction, eGFR, and natriuretic peptide concentration [10].

Anemia is known to be a negative prognostic factor for both heart failure and chronic kidney disease. For heart failure patients, ferric carboxymaltose is the only intravenous iron therapy that current guidelines recommend. Oral iron supplements are not indicated due to their adverse digestive effects and long treatment periods [36].

Regarding anemic patients with non-dialysis CKD, KDIGO guidelines recommend iron therapy in order to reduce ESA doses or to increase Hb levels with a trial of intravenous or oral iron for patients with ferritin ≤500 μg/L and TSAT ≤30% [37].

Silverberg et al. showed that for 179 patients with mild to moderate chronic kidney disease and moderate to severe heart failure, the administration of iron and EPO for the treatment of anemia was associated with a better left ventricular systolic function and an improvement in renal function [23].

Considering that there are physiopathological differences between CKD and HF, it is important to emphasize that CKD patients are already iron-replete. This aspect, as demonstrated in magnetic resonance studies, concludes that lacking available iron is secondary to hepcidin’s blockade of delivering stored iron to cells.

Taking into account all these aspects, the cardiorenal-anemia syndrome must be viewed as a unit with personalized therapeutic strategies adapted to the patient’s profile. Therapies such as intravenous iron, erythropoiesis-stimulating agents, or hypoxia-inducible factor–prolyl hydroxylase domain inhibitors are under consideration in the light of recent studies [20].

### 4.1. Intravenous Iron

Heart failure and anemia have the same symptoms (breathlessness, reduced functional capacity, fatigue) that contribute to poor prognosis. As magnetic resonance imaging shows, the total myocardial iron is lower in heart failure, intravenous iron therapy is being tested in large trials with beneficial results in reducing mortality and hospitalizations [20].

Intravenous iron therapy ameliorates iron parameters in patients with heart failure who present iron deficiency: serum ferritin <100 mg/L or 100–299 mg/L when transferrin saturation <20%, with or without cardiorenal syndrome or renal disease. In the Ferric Iron in Heart Failure (FERRIC-HF) trial, intravenous iron sucrose was associated with increased transferrin saturation (*p* < 0.05), serum ferritin (*p* < 0.01), NYHA functional class (*p* < 0.01) compared with no treatment but without changes in hemoglobin (Hb ≤ 12.5 g/dL) [38].

Increased levels of hemoglobin, transferrin saturation, and serum ferritin were observed in patients with an eGFR ~64 mL/min per 1.73 m^2^ and chronic heart failure who received intravenous ferric carboxymaltose in the Ferinject Assessment in Patients with Iron Deficiency and Chronic Heart Failure (FAIR-HF) trial [7].

Studies that compared oral to intravenous iron therapy found that in CKD patients, intravenous iron increased Hb levels (11.0 to 12 g/dL), more than oral iron. Also in HF, oral iron had no superiority in increasing Hb or other parameters [19].

There is evidence that oral iron produces changes in the gut microbiota, contributing to an increase in the production of uremic toxins in CKD [11,39,40]. Intravenous iron sucrose 200 mg/wk for 6 weeks in patients with anemia (Hb < 12 g/dL) and cardiorenal syndrome increased Hb levels from 10.6 to 11.9 g/dL (*p* < 0.001), results that are similar to the combined treatment with epoetin-b (Hb from 10.2 to 12.4 g/dL; *p* < 0.001) studies, as suggested in [41]. Therefore, i.v. iron therapy without ESAs appears to be sufficient for the management of patients with CRS and anemia. Kidney Disease: Improving Global Outcomes (KDIGO) Anemia Work Group [13].

For patients on hemodialysis, the administration of iron via the dialysate fluid could represent a method for iron deficiency treatment. Studies suggest that the use of ferric pyrophosphate citrate increases hemoglobin levels compared with placebo [42,43].

The FIND-CKD study, one of the largest global multicenter studies that compared intravenous iron therapy to oral administration in CKD-anemia non-dialysis populations and without ESA, showed that the total number of patients whose hemoglobin levels increased by more than 1 g/dL was superior for those who received ferric carboxymaltose (56.9% and 32.1% oral iron, *p* < 0.01) [44]. Even in people without CKD, excessive iron treatment can have negative consequences for life prognosis [45]. 

Using MRI, Rostoker et al. demonstrated that patients with hemodialysis with a ferritin level >290 ng/mL had iron deposits in the liver [46]. Numerous studies have recommended various criteria for iron management, taking into account the fact that frequent intravenous iron administration could increase oxidized albumin and serum ferritin levels [47]. Comparing high-dose intravenous iron (400 mg/month) with low-dose (100 mg/month), Macdougall et al. found that for hemodialysis patients, 400 mg/month iron reduced blood transfusion therapy and improved the death rate [48]. Iron therapy represents a priority even at values of TSAT 30% and serum ferritin 500 ng/mL, as the KDIGO guideline recommends [13]. 

### 4.2. Erythropoiesis-Stimulating Agents

Erythropoietin (EPO) is a 30.4 kDa glycoprotein hormone expressed in the lungs, spleen, brain tissues, and liver, but the principal place of synthesis is represented by peritubular fibroblasts of the renal cortex [49].

ESA has different pharmacodynamic and pharmacokinetic characteristics. The differences between short-acting and long-acting ESAs were shown in the Japanese Registry of Dialysis Study, which highlighted that patients treated with long-acting ESAs presented a 20% higher risk compared to those who received short-acting ESAs for mortality of any cause [50]. Animal studies show that EPO could also reduce apoptotic cell death secondary to coronary reperfusion or ischemia [51].

In heart failure, chronic inflammation represents an important cause of erythropoietin resistance [52]. In the RED-HF (Reduction of Events by Darbepoetin Alfa in Heart Failure) trial, 2278 patients with HF (LVEF ≤ 40%) and hemoglobin levels between 9 and 12 g/dL were randomized to placebo or treatment with darbepoetin alfa, and both groups received treatment with oral or intravenous iron. There were observed increased rates of ischemic stroke (41 (4.5%) vs. 32 (2.8%); *p* = 0.03) and thrombotic events (153 (13.5%) vs. 114 (10.0%); *p* = 0.009) in the darbepoetin alfa group [53].

The consensus regarding the clinical use of ESA highlighted a hemoglobin level inferior to 13 g/dL, or, as other guidelines recommend, a hemoglobin target of 11.5–12 g/dL [13,54].

Patients with non-dialysis-dependent CKD and type-2 diabetes with poor response to initial ESA treatment are prone to increased rates of adverse cardiovascular events (HR 1.31, 95% CI 1.09–1.59) and elevated risks of all-cause death (HR 1.41, 95% CI 1.12–1.78), as the TREAT trial shows [55].

From the first two recombinant human erythropoietins, epoetin alfa and beta, to darbepoetin alfa and methoxy polyethylene glycol-epoetin beta, all developed molecules activate the erythropoietin receptor [56]. 

The hyporesponse or resistance to an erythropoiesis-stimulating agent (ESA) represents the incapacity to reach the target hemoglobin level or the necessity to increase the dose of an ESA to reach the target Hb concentration. The National Institute for Health and Care Excellence (NICE) hyporesponse definition indicates a dose of ESA greater than the maximum initial recommended dose (50–100 units/kg/thrice weekly). For darbepoetin alfa a 0.45 μg/kg/week as a starting dose represents a 3-fold greater than the starting dose and relates to a hyporesponse as NICE indicates [57].

Silverberg et al. showed that ESA used for the treatment of anemia in patients with cardiorenal syndrome improved the ejection fraction (EF), eGFR, patient symptoms, NYHA classification, and shortened the hospitalization period [58]. The regression of left ventricular mass index (LVMI) in patients with CKD was confirmed by Ayus et al. [59]. 

Maintaining hemoglobin levels above 12 g/dL with an early intervention with ESA was demonstrated to reduce the LVMI in pre-dialysis patients in Japan and delay the progression of CKD [60,61,62]. Due to the high risk of cardiovascular events and mortality, for CKD patients, high doses of ESA in order to obtain a level of hemoglobin >11 g/dL are not indicated [63]. The CHOIR study shows that for patients with CKD, low doses of epoetin alfa and a target hemoglobin level >11.5 g/dL or >12.7 g/dL diminished the risk of heart failure, myocardial infarction, stroke, and death. On the other hand, patients with CKD, high exposure to the ESA, and the lowest hemoglobin levels achieved (≤11.5 g/dL), presented an increased risk of the composite end point [64].

Regarding patients with cardiorenal anemia syndrome (CRAS), treatment with epoetin-beta for 50 weeks shows significant increases for both hematocrit and hemoglobin levels and for C-terminal fibroblast growth factor 23, which contributes to the increase in cardiovascular risk and mortality [65]. According to recent research, severe CKD and anterior iron therapy are predictors of the start of ESA medication, which only benefits 10% of patients with CRAS [66]. 

### 4.3. HIF-PH Inhibitors

The hypoxia-inducible factor (HIF)–prolyl hydroxylase domain (PHD) pathway intervenes in the regulation of the cellular response to hypoxia and is implicated in diseases such as ischemic diseases, cancer, anemia, and pulmonary arterial hypertension [67]. 

Hypoxia-inducible factor–prolyl hydroxylase domain inhibitors (HIF-PHIs) act on erythropoiesis by increasing endogenous EPO production, reducing hepcidin levels, and having beneficial effects on iron metabolism. By reducing the need for intravenous iron supplementation and increasing transferrin levels and iron-binding capacity, HIF-PHIs are efficacious in patients with CKD (non-dialysis- and dialysis-dependent), where they improve and maintain the hemoglobin level [68]. HIF-PH inhibitors protect organs from ischemic injuries and improve prognosis in patients who are on high doses of ESA [69]. 

It is also known from the proven efficacy of gliflozines that by stimulating hypoxia-inducible factors, HIF-1 induces expression of hemoxygenase 1, a tissue-protective gene [70], and HIF-2 activation enhances erythropoietin expression [71] and release from renal interstitial cells, which, together with the diuretic effect of the drugs, may contribute to the clinically observed modest increase in hematocrit and hemoglobin in HF patients [72].

Four HIF-PHIs have been described with different pharmacodynamic and pharmacokinetic characteristics: daprodustat, roxadustat, molidustat, and vadadustat [73]. 

The use of HIF-PH inhibitors for anemia treatment in patients with both HF and CKD is rational due to their capacity to increase hemoglobin without high levels of EPO. It is known that the chronic inflammatory status is a common characteristic in HF and CKD and is responsible for ESA hyporesponsiveness in this category of patients, who require a high dose of ESA to obtain an EPO level that could increase hemoglobin. However, there is evidence that high doses of ESA increase mortality in patients with CKD and are associated with a poor prognosis for those with HF. The co-administration of HIF-PHIs and oral iron could be a method to improve iron deficiency in HF patients [74].

Roxadustat, the first-in-class compound, targets all three HIF-PHDs. It is administered orally, with a half-life of 12–15 h, and is metabolized by phase I oxidation via cytochrome P450 (CYP) 2C8 and phase II conjugation via uridine diphosphate (UDP)-glucuronosyltransferase 1–9 (Table 1) [75].

Recent studies showed that roxadustat could reduce ACE2 expression and mRNA in several cells of a mouse model lung tissue, contributing to the inhibition of SARS-CoV-2 and being a potential tool for the prevention and treatment of COVID-19. Another effect of roxadustat reported by Zhu et al. is the improvement of wound healing in diabetic rats [76]. Roxadustat diminishes total cholesterol, low-density lipoprotein, high-density lipoprotein cholesterol, triglycerides, and very-low-density lipoprotein cholesterol, as Chen and colleagues demonstrated [77]. Compared to the epoetin alfa group, adverse effects are more common in the roxadustat group (14.2% vs. 10%), with a percentage of 3.5% for vascular and cardiac disorders [78,79,80]. Pulmonary arterial hypertension, arteriovenous fistula thrombosis, and vascular disease are several effects of roxadustat treatment shown in clinical trials [81]. Possible adverse effects of HIF-PH inhibitors include the occurrence of retinal disorders, hypertension or thrombosis, as reported in roxadustat versus darbepoetin alfa in the safety analysis of pooled phase-3 trials in hemodialysis patients. The risk of thrombosis seems relatively increased in cases where the administered HIF-PH dose effect runs over the desired hemoglobin concentration [82].

Several studies have established the beneficial role of daprodustat in the management of renal anemia and its non-inferiority to erythropoiesis-stimulating agents. Tsubakihara et al. demonstrated an increase in hemoglobin level (10~12 g/dL) in the ESA-naive population using daprodustat [83]. Daprodustat was approved for use in Japan and has demonstrated promising results in phase II studies for anemia management in both dialysis-dependent and non-dialysis-dependent patients [84].

Vadadustat inhibits all three PHDs and was approved in 2020 in Japan for the treatment of anemia secondary to CKD. In the phase-3 PRO2TECT clinical trial, vadadustat did not fulfil the pre-specified non-inferiority criteria for non-fatal stroke, non-fatal myocardial infarction, or all-cause death [85]. A phase-2 study with 210 pre-dialysis stage patients showed that in the group that received vadadustat, the cystatin-C concentration was lower than in the placebo group (34.9 ng/mL (n = 138) vs. 298.2 ng/mL (n = 72)), highlighting the renal-protective effect of vadadustat [86].

Molidustat: for pre-dialysis patients, studies showed a reduction in ferritin, hepcidin, and LDL cholesterol levels, with an improvement in renal anemia in the hemodialysis population as well [48,84]. Only mild side effects were reported: a few cases of pulmonary hypertension elevation, thrombosis/embolism, and hyperkalemia [18].

Enarodustat, a newly approved HIF-PH inhibitor, as studies indicate, shows efficacy and safety in correcting hemoglobin in anemic patients with CKD. Comparing to ESAs, enarodustat improved iron utilization without hyper-producing EPO. Phase III comparative studies (SYMPHONY ND and HD studies) showed that enarodustat is non-inferior to darbepoietin alfa in reducing hepcidin levels and controlling hemoglobin, with a total iron-binding capacity higher for enarodustat [69]. 

Desidustat, another hypoxia-inducible factor–prolyl hydroxylase (HIF-PH) inhibitor, was approved in March 2022 in India for CKD patients on dialysis and non-dialysis-dependent. It is also used for the treatment of anemia induced by chemotherapy and anemia associated with COVID-2019 infections. In rat models, desidustat decreased liver hepcidin levels and increased plasma EPO and reticulocytes. In the phase-3 DREAM-ND study, patients with CKD (non-dialysis-dependent) were randomized to receive desidustat or darbepoetin alfa for 24 weeks, with no inferiority in the desidustat group regarding the treatment of anemia (hemoglobin 7.0–10 g/dL). The same findings were shown in the Phase-3 DREAM-D Study, where desidustat was noninferior to epoetin alfa (hemoglobin 8.0–11.0 g/dL) in CKD dialysis-dependent patients [87].

The use of HIF stabilizers in clinical practice according to Japanese Society of Nephrology (JSN) are: cardiorenal-anemia syndrome, ESA-resistant anemia, and MIA syndrome. The appropriate choice of anemia therapy, either ESA or HIF represents a correct option together with iron therapy, contributing significantly to the prevention of CRA syndrome [18].

Several studies showed that SGLT2 inhibitors diminished hypoxia in the kidney cortex. Layton et al. studied the effects of acute and chronic SGLT2 inhibition and demonstrated reduced oxygen consumption and sodium transport in diabetic proximal tubules and nephrons in a rat model.

By controlling mitochondrial oxygen consumption, luseogliflozin inhibits HIF-1α expression, leading to restoration of hypoxia in the intracellular environment, and further promotes HIF-1α proteasomal degradation in human renal proximal tubular epithelial cells [88].

The benefit of SGLT2 inhibition in enhancing HIF 1 and 2 has relevant implication as HIF-PH may have a variable biological response. The HIF-PH class of drugs is based on molecules that differ in T1/2, so they have different activity duration and their effect is strictly limited to the time they are present in the circulation. Importantly, the efficacy of HIF-PH in restoring the hemoglobin level is also linked to the improved iron assimilation in the gut, providing a perspective to restrict the need to add iron supplementation in CKD and perhaps in HF anemia management [89].

**Table 1:** Mechanisms of action, clinical, and pharmacologic properties of hypoxia-inducible factor– prolyl hydroxylase inhibitors.

	Roxadustat	Daprodustat	Vadadustat	Molidustat	Enarodustat	Desidustat
PHD target	all 3 HIF-PHDs	inhibits PHD1 and PHD3	all three PHDs	mainly inhibits PHD2	all 3 HIF-PHDs	
Benefic Effects	- improvement of wound healing in diabetic rats - diminishes total cholesterol - inhibition of SARS-CoV-2 [18,76,81]	- increased - Hb levels in a dose-dependent manner [18,83]	- renal protective effect lowering cystatin-C concentration - increases of Hb levels [18,86]	- reduction in ferritin, hepcidin and LDL cholesterol level - improving of renal anemia for hemodialysis population [18,48,84]	- improved iron utilization without hyper-production of EPO - increases of Hb levels [69]	increased EPO levels and decreased hepcidin and low-density lipoprotein cholesterol (LDL-C) levels, improves EPO-sensitivity by decreasing IL-6, IL-1β, and anti-EPO antibodies [87]
Adverse effects	- Pulmonary arterial hypertension - arteriovenous fistula thrombosis	- Nausea - Hyperkalemia - increased systolic blood pressure - retinal hemorrhage and hypersensitivity (rash, dermatitis, urticaria)	- gastrointestinal (diarrhea and nausea)	- pulmonary hypertension - thrombosis/embolism - hyperkalemia	- hypertension - hyperkalemia - retinal disorders - viral upper respiratory tract infection and gastrointestinal reactions	Pyrexia, vomiting, asthenia, peripheral oedema
Half life (h)	12~15 h	1.3~2.5 h	7~9 h	4~10 h	15 h	6–15 h
Study population	DD (HD/DP) and NDD	DD (HD/DP) and NDD patients with HF and renal anemia	DD (HD/DP) and NDD	DD (HD/DP) and NDD	DD (HD/DP) and NDD	treatment of anemia associated with CKD (DD and NDD), COVID-2019 infections and chemotherapy induced anemia

PHD: prolyl hydroxylase domain protein; HIF: hypoxia-inducible factor; Hb: hemoglobin; LDL: low-density lipoprotein; EPO: erythropoietin; IL-6: interleukin-6; IL-1β: interleukin-1β; DD: dialysis dependent; HD: hemodialysis; DP: peritoneal dialysis; NDD: non-dialysis dependent.

### 4.4. Hepcidin Antagonist

Hepcidin, a regulator of iron homeostasis, may be responsible for erythropoietin resistance by directly inhibiting the proliferation of erythroid progenitors [90]. 

Due to the main site of elimination of hepcidin (the kidney), patients with CKD will have increased levels of hepcidin. Ashby et al. demonstrated that in hemodialysis (HD) patients, the EPO dose is inversely correlated with hepcidin levels [91]. 

Regarding hepcidin levels, anemias can be divided into anemias with high and low hepcidin. While in the first group, the anemia is due to the inhibitory effect of hepcidin on the absorption of iron, leading to iron deficiency anemia, in the second group, the anemia is the cause of the suppression of hepcidin due to an abnormal erythropoiesis [92]. Patients with CRSA have increased levels of hepcidin [7] (Table 2). In an animal chronic renal failure model, it was demonstrated that EPO exerts an inhibitory effect on hepcidin expression [93].

It was observed that there was a reduction in hepcidin levels during EPO treatment in CKD patients, which was associated with an increase in hemoglobin response, a fact that can be used to predict EPO responsiveness in HF patients [91]. In the HF setting, the EPO administration did not prove efficacy on outcome, but the possible benefit linked to the demonstration deserves large-scale trials to examine the effect and safety of anemia treatment in HF patients [94].

Interleukin 6 (IL-6) is a regulator of hepcidin synthesis. In patients with rheumatoid arthritis, the administration of tocilizumab (a humanized anti-IL-6 receptor antibody) reduces Il-6 levels and increases hemoglobin levels [95]. Considering that the Il-6 effect is mediated by the JAK-STAT pathway, it has been demonstrated that JAK2-STAT3 inhibitors improve anemia in animal models by reducing hepcidin levels. Recent studies showed that anti-ferroportin antibodies LY2928057, by preventing the binding of hepcidin to ferroportin, lead to better hemoglobin levels in CKD patients [96]. 

Other possibilities for hepcidin inhibition, as studies suggest, are thiazolidinediones, vitamin D, estrogens, and testosterone. Direct hepcidin inhibitors are those drugs that inhibit hepcidin-binding to ferroportin: anticalin proteins (e.g., PRS-080), guanosine 5’-diphosphate (GDP), monoclonal antibodies (AB 12B9M), or aptamers (e.g., NOX-H94).

PRS080#022 is a polyethylene glycol-bound anticalin protein that could represent a potential tool in anemia treatment for CKD patients. This class of proteins is a novel class of stable, small proteins that transport and store vitamins or hormones in many organisms. Repeated doses of PRS-080#022 in cynomolgus monkeys demonstrated the suppression of hepcidin.

Renders et al. showed that for the CKD group treated with PRS080#022, TSAT and iron levels increased, with maximum values after 19 h and a dose-dependent duration of effect until 72 h post-PRS-080 administration for the highest dose group [97].

**Table 2:** Target therapies for anemic conditions in accordance with the main mechanisms involved.

Inflammatory status	Hepcidin antagonists anti-IL-6 receptor antibody [91,95]
Iron deficiency	Intravenous iron therapy [38,42,43]
Hypoxic environment	Erythropoiesis-stimulating agents HIF-PH inhibitors SGLT2 [58,67,88]

HIF-PH inhibitors: hypoxia-inducible factor–prolyl hydroxylase inhibitors; IL-6: interleukin-6; SGLT2: sodium-glucose cotransporter-2.

## 5. Future Perspectives

Multiple trials and studies are necessary to evaluate the beneficial and adverse reactions of all therapeutic agents regarding anemia treatment in both HF and CKD patients. Comparisons between ESA and PHD inhibitors were made on CKD patients (dialysis- and non-dialysis-dependent), but did not include the vast majority of the heart failure population. Data from both pathological conditions are needed in order to obtain a specific treatment plan for cardiorenal anemia syndrome. 

## 6. Conclusions

Anemia complicates both heart failure and chronic kidney disease and is a negative prognostic factor. Treating anemia in the context of the cardiorenal syndrome could be challenging due to the multiple interactions and pathophysiologic complexity. From iron therapy, ESA therapy, to hepcidin inhibitors, or HIF-PH inhibitors, beyond the therapeutic targets, there is a reduction in complications and decreased mortality rates. 

Several drugs that are under clinical evaluation have shown good results in terms of safety and efficacy, but more data are needed to confirm their utility in the CRA syndrome therapeutic plan.

## Data Availability

Not applicable.
